# Vitamin D and Weight Change: A Mendelian Randomization, Prospective Study

**DOI:** 10.3390/ijms231911100

**Published:** 2022-09-21

**Authors:** Pollyanna Patriota, Serge Rezzi, Idris Guessous, Pedro Marques-Vidal

**Affiliations:** 1 Swiss Nutrition and Health Foundation, 1066 Epalinges, Switzerland; 2 Division of Primary Care Medicine, Department of Primary Care Medicine, Geneva University Hospitals, 1205 Geneva, Switzerland; 3 Department of Medicine, Internal Medicine, Lausanne University Hospital and University of Lausanne, 46 rue du Bugnon, 1011 Lausanne, Switzerland

**Keywords:** vitamin D, weight change, mendelian randomization, prospective study, Switzerland

## Abstract

The association between 25-hydroxyvitamin D and 5-, 10-, or 15-year weight change were assessed in a population-based, prospective study conducted in Lausanne, Switzerland. Data from the first (2009–2012, N = 3527, 51.3% women), second (2014–2017, N = 3237, 53.8% women), and third (2018–2021, N = 2567, 54.2% women) follow-ups were used. A weighted genetic risk score (GRS) of 115 SNPs associated with vitamin D levels was constructed. At baseline, the GRS correlated positively with 25-hydroxyvitamin D levels based on a Spearman rank correlation and 95% confidence interval: 0.198 (0.166; 0.231), *p* < 0.001; and with body mass index: 0.036 (0.004; 0.068), *p* = 0.028. No association was found between quartiles of GRS and weight changes at 5, 10, or 15 years: multivariate-adjusted weight changes ± SEM at 5-years follow-up were 1.39 ± 0.17, 1.13 ± 0.17, 1.24 ± 0.17, and 1.00 ± 0.17 kg for the first to the fourth quartile of the GRS, respectively (*p* = 0.401). Two-step linear regression showed a significant but clinically meaningless association between GRS-derived vitamin D and weight change at 5- and 15-years: slope and 95% confidence interval for a 5 nmol/L increase in GRS-derived 25-hydroxyvitamin D levels: 0.082 (0.013; 0.150) and 0.130 (0.018; 0.243) kg, respectively. We conclude that there is little association between genetically determined 25-hydroxyvitamin D levels and weight gain.

## 1. Introduction

The associations between vitamin D levels and obesity are controversial [1]. Obesity is associated with low vitamin D levels, but vitamin D supplementation does not appear to reduce weight [1]. Still, most studies focusing on vitamin D levels were of observational nature and are prone to residual confounding, a condition that can be prevented by Mendelian randomization (MR). MR is based on the principle that genotypes are randomly distributed during meiosis and thus unaffected by reverse causation bias [2]. Although several MR studies use individual alleles as instruments, the use of genetic risk scores (GRS) has become increasingly important in MR studies [3]. In order to be applied in MR, the GRS has to fulfil several conditions, namely that the GRS is: (1) linked to the exposure (in this case, vitamin D levels); (2) independent of the outcome (weight change); and (3) not associated with confounders [2]. A GRS for vitamin D has been used to identify a positive association between vitamin D and free-fat mass [4], while a MR using a 4 SNP GRS found no association between vitamin D and body mass index (BMI) [5], a finding also reported elsewhere [6]. Conversely, an inverse association between genetically-instrumented BMI and vitamin D was reported [5]. Still, to our knowledge, no study ever assessed the association between vitamin D levels and weight gain using MR.

Hence, this study aimed at assessing the associations between the GRS for 25-hydroxyvitamin D levels and weight changes at 5-, 10-, and 15-years after baseline using data from a prospective, population-based study. We also assessed the effect of genetically-instrumented BMI on 25-hydroxyvitamin D.

## 2. Results

### 2.1. Characteristics of the Retained Participants

For the analyses, excluding participants receiving vitamin D supplementation for a specific reason, the selection procedure of the participants for follow-ups 1, 2, and 3 is indicated in Figure S1, and the comparison between included and excluded participants is indicated in Table S1. Excluded participants were older, less frequently Swiss, had a higher BMI, a higher waist, and lower baseline vitamin D levels than included ones.

For the analyses excluding participants taking any vitamin supplement, the selection procedure of the participants for follow-ups 1, 2, and 3 is indicated in Figure S2, and the comparison between included and excluded participants is indicated in Table S2. Excluded participants were older, less frequently Swiss, had a higher BMI, a higher waist, and lower baseline vitamin D levels than included ones.

### 2.2. Association between Genetically-Determined Vitamin D, 25-Hydroxyvitamin D Levels and Changes in Anthropometric Markers

**Figure 1:**
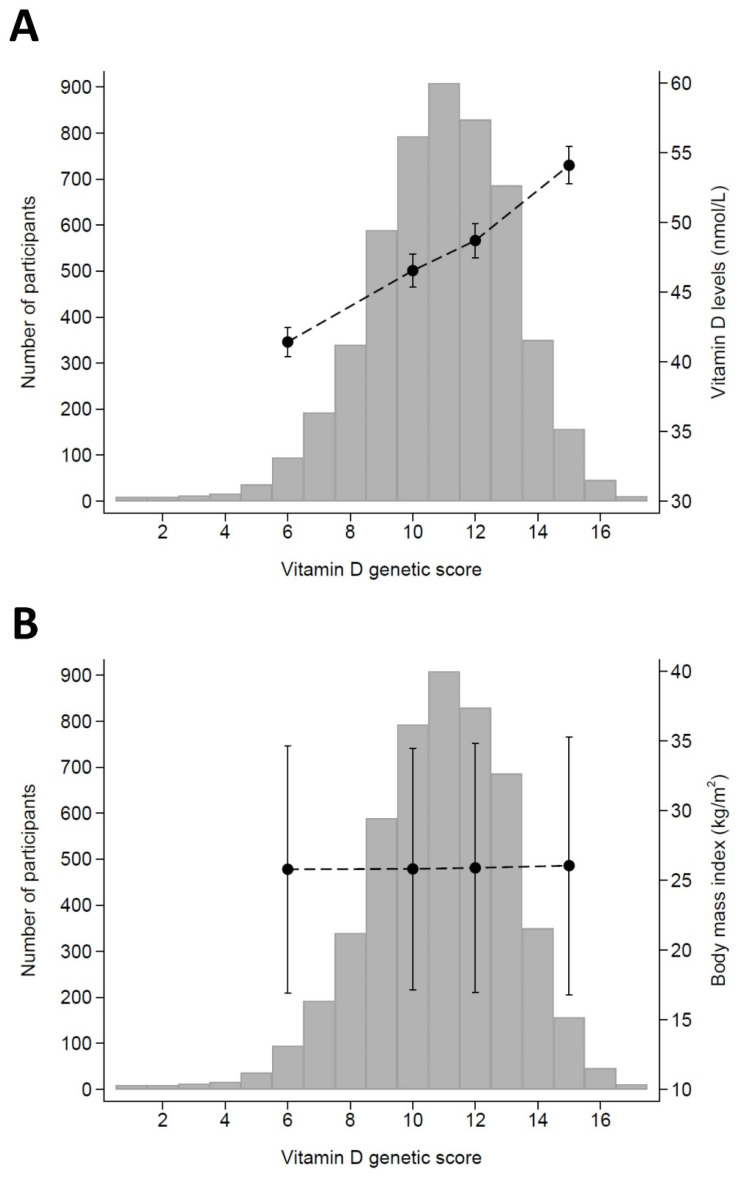
Association between the genetic risk score for vitamin D and 25-hydroxyvitamin D levels (**panel A**) and body mass index (**panel B**), CoLaus study, Lausanne, Switzerland. The *X*-axis represents the genetic risk score values, the bars represent the number of participants, and the dots represent 25-hydroxyvitamin D (**panel A**) or body mass index (**panel B**) values and corresponding 95% confidence intervals.

After excluding participants receiving vitamin D supplementation for a specific reason, the associations between GRS and vitamin D levels and between GRS and BMI at baseline are presented in Figure 1 panel A,B, respectively. The GRS was positively associated with 25-hydroxyvitamin D levels based on a Spearman rank correlation and 95% CI: 0.198 (0.168; 0.228), *p* < 0.001; while the correlation with BMI and waist circumference was smaller: Spearman rank correlation = 0.036 (0.003; 0.069), *p* = 0.028, and 0.037 (0.003; 0.071), *p* = 0.032, respectively. Restricting the analysis to participants devoid of any vitamin supplement led to similar findings: Spearman rank correlation (95% CI) between the GRS and 25-hydroxyvitamin D levels = 0.200 (0.168; 0.233), *p* < 0.001; between the GRS and BMI = 0.029 (−0.005; 0.064), *p* = 0.097; and between the GRS and waist circumference = 0.034 (−0.001; 0.068), *p* = 0.058.

The results for weight change according to quartiles of GRS are summarized in Table 1, after excluding participants receiving vitamin D supplementation for a specific reason, and in Table S3, after excluding participants taking any vitamin supplement. In both conditions, no associations were found between quartiles of GRS and weight or waist changes. Similarly, no specific sex effect was found (Table S4).

The results are expressed as the mean ± standard deviation for bivariate analyses and as the multivariate-adjusted mean ± standard error for multivariate analyses. Analyses were conducted using ANOVA and multivariate analyses adjusted for age, gender, nationality, smoking categories, month of vitamin D assessment, and the first five principal components of the genetic assessment. The results of the MR for weight change at 5.6-, 10.7-, and 14.5-years follow-up are summarized in Table 2, after excluding participants receiving vitamin D supplementation for a specific reason, and in Table S5, after excluding participants taking any vitamin supplement. A positive association with weight change was found at 5 and 15 years, but not at 10 years follow-up (Table 2), and similar results were found after exclusion of participants taking any vitamin supplements (Table S5). No association was found with waist change (Table 2 and Table S5).

### 2.3. Association between Genetically-Determined Body Mass Index and 25-Hydroxyvitamin D Levels

After excluding participants receiving vitamin D supplementation for a specific reason, the GRS for BMI was positively associated with BMI levels at baseline: Spearman rank correlation and 95% CI = 0.071 (0.044; 0.098), *p* < 0.001; while the negative correlation with 25-hydroxyvitamin D levels did not reach statistical significance: −0.006 (−0.033; 0.021), *p* = 0.673. The results of the MR showed that a one-unit increase in genetically-determined BMI was associated with a decrease in 25-hydroxyvitamin D levels: slope and 95% CI = −9.25 (−11.9; −6.56) nmol/L. No difference in 25-hydroxyvitamin D levels was found between quartiles of GRS for BMI: multivariate-adjusted mean±standard deviation = 49.0 ± 0.5, 48.4 ± 0.5, 49.0 ± 0.5, and 47.6 ± 0.5 nmol/L for the first, second, third, and fourth quartile, respectively, *p* for trend = 0.162.

## 3. Discussion

In this population-based study, no association was found between quartiles of a vitamin D genetic risk score and weight or waist change at 5.6, 10.7, and 14.5 years. A positive association was found between genetically-determined 25-hydroxyvitamin D levels and weight change, but the changes were small and of little if no clinical significance. No association between genetically-determined vitamin D levels and waist change was found. The inverse association between genetically-determined BMI and 25-hydroxyvitamin D was confirmed.

### 3.1. Association between Genetically-Determined Vitamin D, 25-Hydroxyvitamin D Levels, and Changes in Anthropometric Markers

A small, positive association was found between vitamin D GRS and anthropometric markers at baseline. Our findings contradict a previous review, where no association between vitamin D genetic markers and obesity in Europeans was found [7]. A possible explanation is that the previous review focused on individual vitamin D metabolism-related SNPs, while in this study we used a GRS derived from 115 different SNPs. Similarly, two previous MR studies concluded on the absence of association between single SNPs [8] and two GRS for vitamin D and obesity levels using a cross-sectional design [5]. Again, a possible explanation is that the GRS for vitamin D in the last study [5] consisted of only two SNPs each, which might not be enough to fully encompass the genetic determinants of vitamin D levels. A recent MR found a positive association between vitamin D and fat-free mass [4], but no information regarding fat mass was provided. Still, the findings could be in line with ours, as an increased fat-free mass could eventually lead to an increase in BMI. A large MR study using a 138-SNP GRS found no association between genetically-determined vitamin D levels and several anthropometric markers [9]. Finally, our results do not confirm the negative association between vitamin D levels and body mass index [10], suggesting that this association might be due to the sequestration of vitamin D by fat deposits in overweight/obese people [11], rather than to be causal. Overall, our results suggest that vitamin D levels might be associated with obesity traits at a cross-sectional setting, but that the strength of the association is low.

Conversely, no association was found between quartiles of the vitamin D GRS and weight change, and a small, clinically irrelevant increase in weight (0.083 kg weight for an increase in 5 nmol/L vitamin D) was found. Our findings thus replicate those from a cross-sectional MR study [9], wherein no association was found between genetically-determined vitamin D and all anthropometric markers considered. They also replicate our previous study where no association between baseline 25-hydroxyvitamin D levels and weight changes were found [12]. Conversely, our findings do not confirm those reported in prospective studies, where high vitamin D levels were found to be protective against obesity risk [13,14]. Interestingly, one study reported a positive association between vitamin D insufficiency and risk of abdominal obesity in both genders, while the association with weight gain was found in men only [15]. Overall, our results do not confirm those from prospective studies, and further MR studies are needed to confirm our findings.

### 3.2. Association between Genetically-Determined Body Mass Index and 25-Hydroxyvitamin D Levels

The GRS for BMI was positively associated with BMI levels, while the negative association with 25-hydroxyvitamin D levels did not reach statistical significance. Our results partly replicate those of Vimaleswaran et al. [5], where a significant negative association between the BMI GRS and vitamin D levels was also reported. A likely explanation is the smaller sample size of our study (N = 5374, compared to N = 42,024 of the previous study), which would lead to a lower statistical power. Importantly, the genetically-determined BMI was significantly and negatively associated with 25-hydroxyvitamin D levels, thus replicating the findings of the previous study [5]. The reasons for the lower vitamin D levels among people with a high BMI are still a matter of debate, wherein dilution in a large pool of fat mass [16] changes in parathormone metabolism [17], reductions in hepatic 25-hydroxylase enzyme activity [18], or differences in lifestyle, such as low physical activity and inadequate dietary intake [19], are likely hypotheses. Overall, our results indicate that increased BMI is associated with lower 25-hydroxyvitamin D levels.

### 3.3. Strengths and Limitations

This study is one of the very few assessing the association between vitamin D and weight/waist changes using MR. The study relied on the most recent data on SNPs related with vitamin D levels and on a large, population-based sample followed for a long period.

This study has some limitations. Firstly, the sample included only Caucasian subjects living in a single city, and it is unknown if the GRS values will be similar in other populations with differing ethnicities, as suggested previously [7]. Hence, the generalizability of the findings should be tested in other settings. Secondly, vitamin D levels were only assessed once, and it could have been interesting to monitor the associations between changes in vitamin D levels and changes in anthropometric markers.

## 4. Materials and Methods

### 4.1. Participants

The CoLaus|PsyCoLaus (www.colaus-psycolaus.ch (accessed on 1 January 2020)) is a single-center, prospective cohort study established in 2003 following a sample of the inhabitants of the city of Lausanne (Switzerland), aged 35 to 75 years at baseline, every 5 years [20]. In each survey, participants answered questionnaires, underwent a clinical examination, and blood samples were drawn for analyses. The baseline study was conducted between June 2003 and May 2006. The first follow-up was performed between April 2009 and September 2012, the second follow-up between May 2014 and April 2017, and the third follow-up between April 2018 and May 2021. Median follow-up time was 5.4 (average 5.6, range 4.5–8.8) years for the first follow-up, 10.7 (average 10.9, range 8.8–13.6) years for the second follow-up, and 14.5 (average 14.6, range 13.2–17.3) for the third follow-up.

### 4.2. Genotyping

Genome-wide genotyping was performed using the Affymetrix 500K SNP array. Nuclear DNA was extracted from the whole blood of all participants. Genotypes were called using BRLMM (www.affymetrix.com/support/technical/whitepapers/brlmm_whitepap (accessed on 1 January 2020)). Duplicate individuals, and first- and second-degree relatives, were identified and removed by computing estimates through pairwise genomic kinship coefficients, using KING [21].

Subjects were excluded from the analysis in case of inconsistency between sex and genetic data, a genotype call rate < 90%, or inconsistencies of genotyping results in duplicate samples. Quality control for SNPs was performed using the following criteria: monomorphic (or with minor allele frequency (MAF) < 1%), call rates < 90%, and deviation from the Hardy-Weinberg equilibrium (HWE) (*p* < 1 × 10^−6^).

Phased haplotypes were generated using SHAPEIT2 [22,23]. Imputation was performed using minimac3 [24] and the Haplotype Reference Consortium (HRC version r1.1) [25] hosted on the Michigan Imputation Server [24]. The principal components analysis was performed on the genotypes to assess genetic variation [26,27].

We selected 143 SNPs associated with vitamin D from a recent GWAS [28], of which 115 (80%) were available in the CoLaus database. Those 115 SNPs were used to compute a weighted genetic risk score (GRS) for vitamin D. Weighting was based on the beta values related to each SNP after conditional and joint analyses as reported in the GWAS. The list of SNPs and corresponding beta values is provided in Table S6. Briefly, the GRS was computed as follows: (1) for each SNP, the number of reference alleles was multiplied by the corresponding beta value; and (2) for each participant, the values for each SNP were added.

The GRS for BMI was computed as performed previously [5] using 12 SNPs and no weighting. The list of SNPs is provided in Table S7. The association between genetically-determined BMI and 25-hydroxyvitamin D levels was assessed at baseline.

### 4.3. Vitamin D Assessment

The assessment of 25-hydroxyvitamin D (25OHD) was performed at baseline through an ultra-HPLC tandem-MS system. The calibrators, 3Plus1 Multilevel Serum Calibrator Set 25-OH-Vitamin D_3_/D_2_ (ChromoSystems, Gräfelfing, Germany), were standardized against the National Institute of Standards and Technology 972 reference material. The results were expressed in nanomoles per liter (conversion factor: 1 nmol/L = 0.4006 μg/L). The interday CV% was 4.6% at 40 nmol/L.

### 4.4. Weight and Waist Assessment

Anthropometric measurements were conducted using a standard methodology. Body weight and height were measured with participants barefoot and in light indoor clothes. Body weight was measured in kilograms to the nearest 100 g using a Seca^®^ scale (Hamburg, Germany). Height was measured to the nearest 5 mm using a Seca^®^ (Hamburg, Germany) height gauge. Weight change was defined at each follow-up as the difference between the baseline and the measurement at follow-up and two metrics were used as previously [29]: (1) as a continuous variable and (2) as percentage change relative to the baseline value.

Waist circumference (WC) was measured mid-way between the lowest rib and the iliac crest, and hip was measured at the largest location, using a non-stretchable tape; the average of the two measurements was taken. Waist change was defined at each follow-up as the difference between the baseline and the measurement at follow-up and used as a continuous variable.

### 4.5. Covariates

Nationality was categorized as born in Switzerland or not. Smoking status was self-reported and categorized as never, former, and current. The month corresponding to the measurement of vitamin D was used to adjust for seasonality.

### 4.6. Inclusion and Exclusion Criteria

Participants were considered as eligible if they had vitamin D levels and genetic data at baseline. Participants were excluded if they (1) had no data for weight, (2) reported taking vitamin supplements at baseline, (3) did not participate in follow-up, or (4) reported being on a diet to reduce at follow-up. For exclusion criterion (3), two categories were considered: (a) vitamin D supplementation for a specific disease (i.e., osteoporosis) or (b) any vitamin supplementation.

### 4.7. Statistical Analyses

Analyses were conducted using Stata v.16.1 for Windows (Stata corp, College Station, TX, USA). Descriptive statistics were presented as the number of participants (percentage) for categorical variables and as the mean ± standard deviation or as a median [interquartile range] for continuous variables. Between-group comparisons were performed using chi-square for categorical variables and student’s *t*-test or Kruskal–Wallis test for continuous variables. Bivariate associations between the GRS and other variables were assessed by Spearman rank correlation, and 95% confidence intervals (CI) were computed by bootstrapping. Changes in weight according to quartiles of GRS were assessed by ANOVA for bivariate and multivariate analyses. Multivariate analyses were adjusted for age, gender, nationality, smoking categories, month of vitamin D assessment, and the first five principal components of the genetic assessment. A specific effect of sex was explored by introducing an interaction term between sex and quartiles of GRS.

For MR analysis, the associations between 25-hydroxyvitamin D, as mediated by the GRS, and changes in weight were assessed by 2-step linear regression, using age, gender, nationality, smoking categories, and the first 5 principal components of the genetic assessment as exogenous variables, vitamin D levels as endogenous variable, and the GRS and the month of vitamin D assessment as instrumental variables. A similar approach was used when assessing the association between genetically-determined BMI and 25-hydroxyvitamin D levels.

Two sets of analyses were conducted: after excluding participants taking (a) vitamin D supplementation for a specific disease and (b) any vitamin supplementation. Statistical significance was considered for a two-sided test with *p* < 0.05.

### 4.8. Ethical Statement

The institutional Ethics Committee of the University of Lausanne, which afterwards became the Ethics Commission of Canton Vaud (www.cer-vd.ch (accessed on 1 January 2020)), approved the baseline CoLaus study (reference 16/03). The approval was renewed for the first (reference 33/09), the second (reference 26/14), and the third (reference PB_2018-00040) follow-ups. The approval for the entire CoLaus|PsyCoLaus study was confirmed in 2021 (reference PB_2018-00038, 239/09). The full decisions of the CER-VD can be obtained from the authors upon request. The study was performed in agreement with the Helsinki declaration and its former amendments, and in accordance with the applicable Swiss legislation. All participants gave their signed informed consent before entering the study.

## 5. Conclusions

We found little association between genetically-determined vitamin D levels and weight or waist gain. The positive association with weight gain observed at 5.6- and 14.5-years is statistically significant but clinically meaningless. The negative association between genetically-determined BMI and 25-hydroxyvitamin D levels was confirmed.

## Figures and Tables

**Table 1:** Bivariate and multivariate analysis of 5-, 10-, and 15-year changes in weight according to quartiles of the genetic risk score for vitamin D, CoLaus study, Lausanne, Switzerland.

	First Follow-Up (5.6 Years)		Second Follow-Up (10.7 Years)		Third Follow-Up (14.5 Years)	
	Bivariate	Multivariate	Bivariate	Multivariate	Bivariate	Multivariate
Weight change (kg)						
First	1.46 ± 4.95	1.40 ± 0.17	2.04 ± 6.67	1.91 ± 0.22	1.94 ± 7.34	1.75 ± 0.28
Second	1.11 ± 4.95	1.13 ± 0.16	2.00 ± 6.51	2.05 ± 0.22	2.16 ± 7.29	2.18 ± 0.28
Third	1.22 ± 5.08	1.23 ± 0.17	1.70 ± 6.32	1.71 ± 0.22	1.49 ± 7.65	1.49 ± 0.28
Fourth	0.96 ± 5.02	1.00 ± 0.17	1.32 ± 6.62	1.40 ± 0.22	1.43 ± 7.52	1.61 ± 0.28
*p*-value	0.205	0.379	0.098	0.180	0.225	0.343
Weight change (%)						
First	2.1 ± 6.7	2.0 ± 0.2	2.9 ± 9.1	2.7 ± 0.3	3.0 ± 10.5	2.7 ± 0.4
Second	1.6 ± 6.6	1.6 ± 0.2	2.8 ± 8.8	2.9 ± 0.3	3.1 ± 9.9	3.2 ± 0.4
Third	1.8 ± 6.5	1.8 ± 0.2	2.5 ± 8.2	2.5 ± 0.3	2.3 ± 10.0	2.3 ± 0.4
Fourth	1.4 ± 6.6	1.5 ± 0.2	2.0 ± 8.9	2.1 ± 0.3	2.3 ± 10.2	2.5 ± 0.4
*p*-value	0.210	0.409	0.149	0.248	0.283	0.430
Waist change (cm)						
First	3.8 ± 6.8	3.8 ± 0.2	4.0 ± 7.6	4.0 ± 0.3	4.5 ± 8.3	4.4 ± 0.3
Second	3.1 ± 6.7	3.1 ± 0.2	4.0 ± 7.6	4.0 ± 0.3	4.5 ± 7.9	4.4 ± 0.3
Third	3.4 ± 6.9	3.4 ± 0.2	3.6 ± 7.2	3.6 ± 0.3	4.2 ± 8.5	4.2 ± 0.3
Fourth	3.1 ± 6.9	3.2 ± 0.2	3.6 ± 7.7	3.6 ± 0.3	4.6 ± 8.5	4.6 ± 0.3
*p*-value	0.093	0.168	0.395	0.504	0.869	0.877

**Table 2:** Mendelian randomization results of the association between 25-hydroxyvitamin D levels and 5-, 10-, and 15-year changes in weight, CoLaus study, Lausanne, Switzerland.

	First Follow-Up (5.6 Years)	Second Follow-Up (10.7 Years)	Third Follow-Up (14.5 Years)
Weight change (kg)	0.082 (0.013; 0.150)	0.035 (−0.058; 0.128)	0.130 (0.018; 0.243)
*p*-value	0.019	0.463	0.023
Weight change (%)	0.105 (0.015; 0.195)	0.052 (−0.072; 0.177)	0.168 (0.016; 0.321)
*p*-value	0.022	0.409	0.031
Waist change (cm)	−0.034 (−0.127; 0.060)	0.016 (−0.095; 0.128)	0.098 (−0.033; 0.228)
*p*-value	0.481	0.774	0.143

Results are expressed as slope and 95% confidence interval for an increase in 5 nmol/L vitamin D. Analysis performed using two-step linear regression, using age, gender, nationality, smoking categories, and the first five principal components of the genetic assessment as exogenous variables, vitamin D levels as endogenous variable, and the GRS and the month of vitamin D assessment as instrumental variables.

## Data Availability

The CoLaus|PsyCoLaus cohort data used in this study cannot be fully shared as they contain potentially sensitive patient information. As discussed with the competent authority, the Research Ethic Committee of the Canton of Vaud, transferring or directly sharing this data would be a violation of the Swiss legislation aiming to protect the personal rights of participants. Non-identifiable, individual-level data are available for interested researchers, who meet the criteria for access to confidential data sharing, from the CoLaus Datacenter (CHUV, Lausanne, Switzerland). Instructions for gaining access to the CoLaus data used in this study are available at https://www.colaus-psycolaus.ch/professionals/how-to-collaborate/ (accessed on 1 January 2020).

## Supplementary Materials

The following supporting information can be downloaded at: https://www.mdpi.com/article/10.3390/ijms231911100/s1.
