# Supplementary material for: Vitamin D and Weight Change: A Mendelian Randomization, Prospective Study
Source: Int J Mol Sci. 2022 Sep 21;23(19):11100. doi: 10.3390/ijms231911100 (PMC9569579; doi:10.3390/ijms231911100)
Supplement: Supplementary file 1 [file ijms-23-11100-s001.zip › Table S7_IJMS.pdf]

Table S7. list of SNPs used to compute the genetic risk score for body mass index.

SNP	Chromosome	Position	Reference allele	Gene
rs10913469	1	177913519	C	GNPDA2
rs3101336	1	72751185	C	NEGR1
rs2867125	2	622827	C	TMEM18
rs7647305	3	185834290	C	ETV5
rs10938397	4	45182527	G	SEC16B
rs4074134	11	27647285	T	BDNF
rs10838738	11	47663049	G	MTCH2
rs7138803	12	50247468	A	FAIM2
rs9939609	16	53820527	A	FTO
rs7498665	16	28883241	G	SH2B1
rs17782313	18	57851097	C	MC4R
rs29941	19	34309532	G	KCTD15
