# Supplementary material for: Vitamin D and Weight Change: A Mendelian Randomization, Prospective Study
Source: Int J Mol Sci. 2022 Sep 21;23(19):11100. doi: 10.3390/ijms231911100 (PMC9569579; doi:10.3390/ijms231911100)
Supplement: Supplementary file 1 [file ijms-23-11100-s001.zip › Tables S1-S5_IJMS.pdf]

Supplementary information

Table S1: characteristics of participants included and excluded, only participants taking vitamin D supplementation for a specific disease, by follow-up, CoLaus study, Lausanne, Switzerland.

	First follow-up (5.6 years)			Second follow-up (10.7 years)			Third follow-up (14.5 years)		
	**Included**	**Excluded**	**P-value**	**Included**	**Excluded**	**P-value**	**Included**	**Excluded**	**P-value**
Sample size	3527	3206		3237	3496		2567	4166	
Women (%)	1808 (51.3)	1736 (54.2)	0.018	1742 (53.8)	1802 (51.5)	0.062	1391 (54.2)	2153 (51.7)	0.045
Age (years)	52.9 ± 10.5	52.4 ± 11.0	0.071	52.2 ± 10.4	53.0 ± 11.1	0.002	50.9 ± 9.9	53.7 ± 11.1	<0.001
Swiss born (%)	2385 (67.6)	1646 (51.4)	<0.001	2175 (67.2)	1856 (53.1)	<0.001	1766 (68.8)	2265 (54.4)	<0.001
Smoking (%)			0.016			0.002			<0.001
Never	1437 (40.7)	1295 (40.5)		1333 (41.2)	1399 (40.1)		1097 (42.7)	1635 (39.3)	
Former	1187 (33.7)	996 (31.1)		1093 (33.8)	1090 (31.2)		854 (33.3)	1329 (32.0)	
Current	903 (25.6)	909 (28.4)		811 (25.1)	1001 (28.7)		616 (24.0)	1196 (28.7)	
BMI (kg/m^2)	25.5 ± 4.3	26.2 ± 4.8	<0.001	25.3 ± 4.2	26.2 ± 4.8	<0.001	25.1 ± 4.1	26.2 ± 4.7	<0.001
BMI categories (%)			<0.001			<0.001			<0.001
Underweight	63 (1.8)	45 (1.4)		57 (1.8)	51 (1.4)		46 (1.8)	62 (1.5)	
Normal	1733 (49.1)	1396 (43.6)		1628 (50.2)	1501 (43.0)		1358 (52.9)	1771 (42.6)	
Overweight	1288 (36.5)	1174 (36.7)		1164 (36.0)	1298 (37.2)		883 (34.4)	1579 (37.9)	
Obese	443 (12.6)	588 (18.4)		388 (12.0)	643 (18.4)		280 (10.9)	751 (18.0)	
Waist (cm)	88.5 ± 13.1	89.6 ± 13.6	<0.001	87.9 ± 12.8	90.1 ± 13.7	<0.001	87.2 ± 12.4	90.2 ± 13.7	<0.001
Genetic risk score	1.09 ± 0.23	1.09 ± 0.23	0.906	1.09 ± 0.23	1.09 ± 0.23	0.436	1.09 ± 0.23	1.09 ± 0.23	0.595
Vit. D (nmol/L)	46.8 [31.7−63.0]	42.4 [27.3−60.5]	§<0.001	46.8 [32.1−63.2]	42.6 [27.3−60.2]	§<0.001	47.9 [33.1−64.7]	42.6 [27.4−59.9]	§<0.001

BMI, body mass index; GRS, genetic risk score; Vit, vitamin. Results are presented as number of participants (percentage) for categorical variables and as mean ± standard deviation or as median [interquartile range] for continuous variables. Between-group comparisons performed using chi-square for categorical variables and student's t-test or Kruskal-Wallis test (§) for continuous variables.

Table S2: characteristics of participants included and excluded, only participants taking any vitamin supplementation, by follow-up, CoLaus study, Lausanne, Switzerland.

	First follow-up (5.6 years)			Second follow-up (10.7 years)			Third follow-up (14.5 years)		
	**Included**	**Excluded**	**P-value**	**Included**	**Excluded**	**P-value**	**Included**	**Excluded**	**P-value**
Sample size	3301	3432		3014	3719		2386	4347	
Women (%)	1672 (50.7)	1872 (54.6)	0.001	1602 (53.2)	1942 (52.2)	0.446	1277 (53.5)	2267 (52.2)	0.282
Age (years)	53.0 ± 10.5	52.3 ± 10.9	0.012	52.3 ± 10.4	52.9 ± 11.0	0.012	50.9 ± 9.8	53.6 ± 11.1	<0.001
Swiss born (%)	2240 (67.9)	1791 (52.2)	<0.001	2027 (67.3)	2004 (53.9)	<0.001	1646 (69.0)	2385 (54.9)	<0.001
Smoking (%)			0.029			0.006			<0.001
Never	1352 (41.0)	1380 (40.3)		1254 (41.6)	1478 (39.8)		1029 (43.1)	1703 (39.2)	
Former	1106 (33.5)	1077 (31.4)		1006 (33.4)	1177 (31.7)		786 (32.9)	1397 (32.2)	
Current	843 (25.5)	969 (28.3)		754 (25.0)	1058 (28.5)		571 (23.9)	1241 (28.6)	
BMI (kg/m^2)	25.5 ± 4.3	26 ± 4.7	<0.001	25.4 ± 4.2	26.1 ± 4.7	<0.001	25.2 ± 4.1	26.1 ± 4.7	<0.001
BMI categories (%)			<0.001			<0.001			<0.001
Underweight	60 (1.8)	48 (1.4)		54 (1.8)	54 (1.5)		44 (1.8)	64 (1.5)	
Normal	1595 (48.3)	1534 (44.7)		1499 (49.7)	1630 (43.9)		1251 (52.4)	1878 (43.2)	
Overweight	1220 (37.0)	1242 (36.2)		1090 (36.2)	1372 (36.9)		826 (34.6)	1636 (37.7)	
Obese	426 (12.9)	605 (17.6)		371 (12.3)	660 (17.8)		265 (11.1)	766 (17.6)	
Waist (cm)	88.8 ± 13.1	89.3 ± 13.5	0.119	88.1 ± 12.7	89.8 ± 13.7	<0.001	87.3 ± 12.5	90.0 ± 13.7	<0.001
Genetic risk score	1.09 ± 0.23	1.09 ± 0.23	0.987	1.09 ± 0.23	1.09 ± 0.23	0.717	1.09 ± 0.23	1.09 ± 0.23	0.763
Vit. D (nmol/L)	46.5 [31.6−62.8]	42.9 [27.7−61.0]	§ 0.001	46.7 [32.0−62.9]	43.1 [27.6−60.8]	§<0.001	47.4 [33.0−64.3]	43.0 [27.7−60.7]	<0.001

BMI, body mass index; GRS, genetic risk score; Vit, vitamin. Results are presented as number of participants (percentage) for categorical variables and as mean ± standard deviation or as median [interquartile range] for continuous variables. Between-group comparisons performed using chi-square for categorical variables and student's t-test or Kruskal-Wallis test (§) for continuous variables.

Table S3: bivariate and multivariate analysis of 5-, 10- and 15-year changes in weight according to quartiles of the genetic risk score for vitamin D, CoLaus study, Lausanne, Switzerland.

	First follow-up (5.6 years)		Second follow-up (10.7 years)		Third follow-up (14.5 years)	
	**Bivariate**	**Multivariate**	**Bivariate**	**Multivariate**	**Bivariate**	**Multivariate**
Weight change (kg)						
First	1.45 ± 5.02	1.39 ± 0.17	2.09 ± 6.72	1.95 ± 0.23	2.01 ± 7.50	1.82 ± 0.29
Second	1.03 ± 5.06	1.03 ± 0.17	1.96 ± 6.58	1.99 ± 0.23	2.11 ± 7.38	2.08 ± 0.29
Third	1.24 ± 5.08	1.25 ± 0.17	1.76 ± 6.33	1.79 ± 0.23	1.54 ± 7.73	1.56 ± 0.29
Fourth	0.97 ± 5.00	1.02 ± 0.17	1.26 ± 6.64	1.34 ± 0.23	1.38 ± 7.48	1.58 ± 0.30
P-value	0.206	0.354	0.075	0.171	0.264	0.558
Weight change (kg)						
First	2.1 ± 6.8	2.0 ± 0.2	3.0 ± 9.2	2.8 ± 0.3	3.1 ± 10.7	2.8 ± 0.4
Second	1.5 ± 6.7	1.5 ± 0.2	2.8 ± 8.8	2.8 ± 0.3	3.1 ± 10.0	3.0 ± 0.4
Third	1.9 ± 6.5	1.9 ± 0.2	2.6 ± 8.1	2.7 ± 0.3	2.4 ± 10.1	2.4 ± 0.4
Fourth	1.5 ± 6.6	1.5 ± 0.2	1.9 ± 8.9	2.0 ± 0.3	2.2 ± 10.1	2.4 ± 0.4
P-value	0.182	0.325	0.110	0.219	0.301	0.639
Waist change (cm)						
First	3.8 ± 6.8	3.7 ± 0.2	4.1 ± 7.6	4.1 ± 0.3	4.5 ± 8.4	4.5 ± 0.3
Second	3.0 ± 6.7	3.0 ± 0.2	3.9 ± 7.6	3.9 ± 0.3	4.4 ± 7.9	4.3 ± 0.3
Third	3.4 ± 6.9	3.4 ± 0.2	3.6 ± 7.2	3.7 ± 0.3	4.2 ± 8.7	4.2 ± 0.3
Fourth	3.1 ± 7.0	3.1 ± 0.2	3.5 ± 7.8	3.5 ± 0.3	4.4 ± 8.5	4.5 ± 0.3
P-value	0.086	0.164	0.365	0.501	0.900	0.927

Results are expressed as mean ± standard deviation for bivariate analyses and as multivariate-adjusted mean ± standard error for multivariate analyses. Analyses conducted using ANOVA; multivariate analyses adjusted for age, gender, nationality, smoking categories, month of vitamin D assessment, and the first five principal components of the genetic assessment.

Table S4: results of the analysis of interaction between gender and quartiles of the genetic risk score for vitamin D, CoLaus study, Lausanne, Switzerland.

	**First follow-up (5.6 years)**	**Second follow-up (10.7 years)**	**Third follow-up (14.5 years)**
Weight change (kg9			
Excluding vitamin D supplements only	0.535	0.059	0.808
Excluding all vitamin supplements	0.487	0.132	0.901
Weight change (%)			
Excluding vitamin D supplements only	0.757	0.126	0.862
Excluding all vitamin supplements	0.640	0.224	0.938
Waist change (cm)			
Excluding vitamin D supplements only	0.163	0.054	0.280
Excluding all vitamin supplements	0.105	0.100	0.431

Results are expressed as p-value of the interaction term between gender and quartiles of the genetic risk score for vitamin D. Analyses conducted using ANOVA adjusted for age, nationality, smoking categories, month of vitamin D assessment, and the first five principal components of the genetic assessment.

Table S5: mendelian randomization results of the association between vitamin D levels and 5-, 10- and 15-year changes in weight, CoLaus study, Lausanne, Switzerland. Participants taking any vitamin supplement were excluded.

	**First follow-up (5.6 years)**	**Second follow-up (10.7 years)**	**Third follow-up (14.5 years)**
Weight change (kg)	0.087 (0.015; 0.159)	0.021 (−0.076; 0.118)	0.112 (−0.006; 0.231)
P-value	0.018	0.668	0.063
Weight change (%)	0.113 (0.018; 0.207)	0.029 (−0.100; 0.158)	0.141 (−0.020; 0.301)
P-value	0.019	0.658	0.086
Waist change (cm)	−0.034 (−0.131; 0.064)	0.008 (−0.107; 0.124)	0.091 (−0.047; 0.229)
P-value	0.496	0.888	0.195

Results are expressed as slope and (95% confidence interval) for an increase in 5 nmol/L vitamin D. Analysis performed using 2-step linear regression, using age, gender, nationality, smoking categories and the first five principal components of the genetic assessment as exogenous variables, vitamin D levels as endogenous variable, and the GRS and the month of vitamin D assessment as instrumental variables.
