# Supplementary figures and images for: Vitamin D and Weight Change: A Mendelian Randomization, Prospective Study
Source: Int J Mol Sci. 2022 Sep 21;23(19):11100. doi: 10.3390/ijms231911100 (PMC9569579; doi:10.3390/ijms231911100)

SPECIFIC

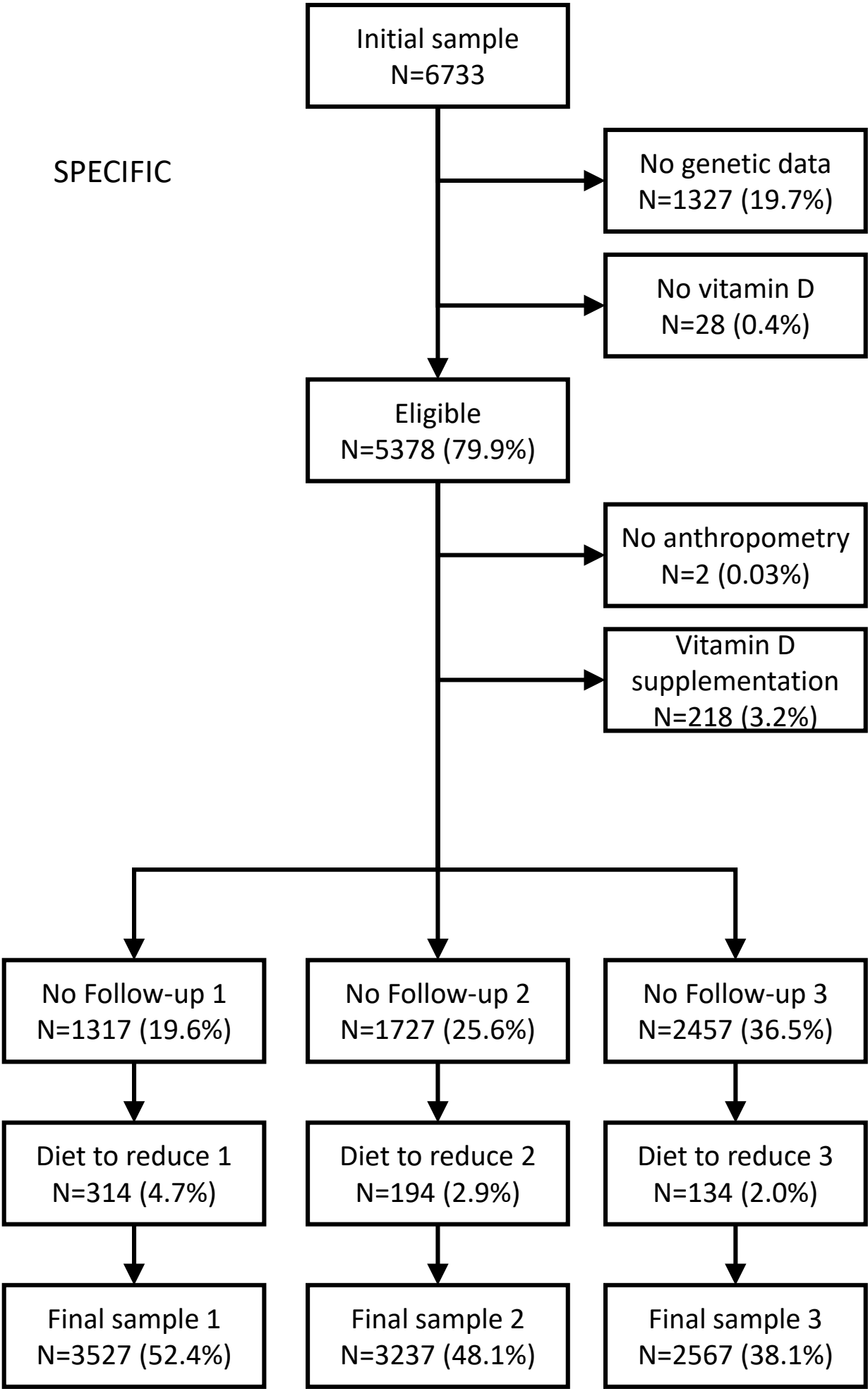

Supplement: Supplementary file 1 [file ijms-23-11100-s001.zip › Figure S1_IJMS.pdf]

ALL

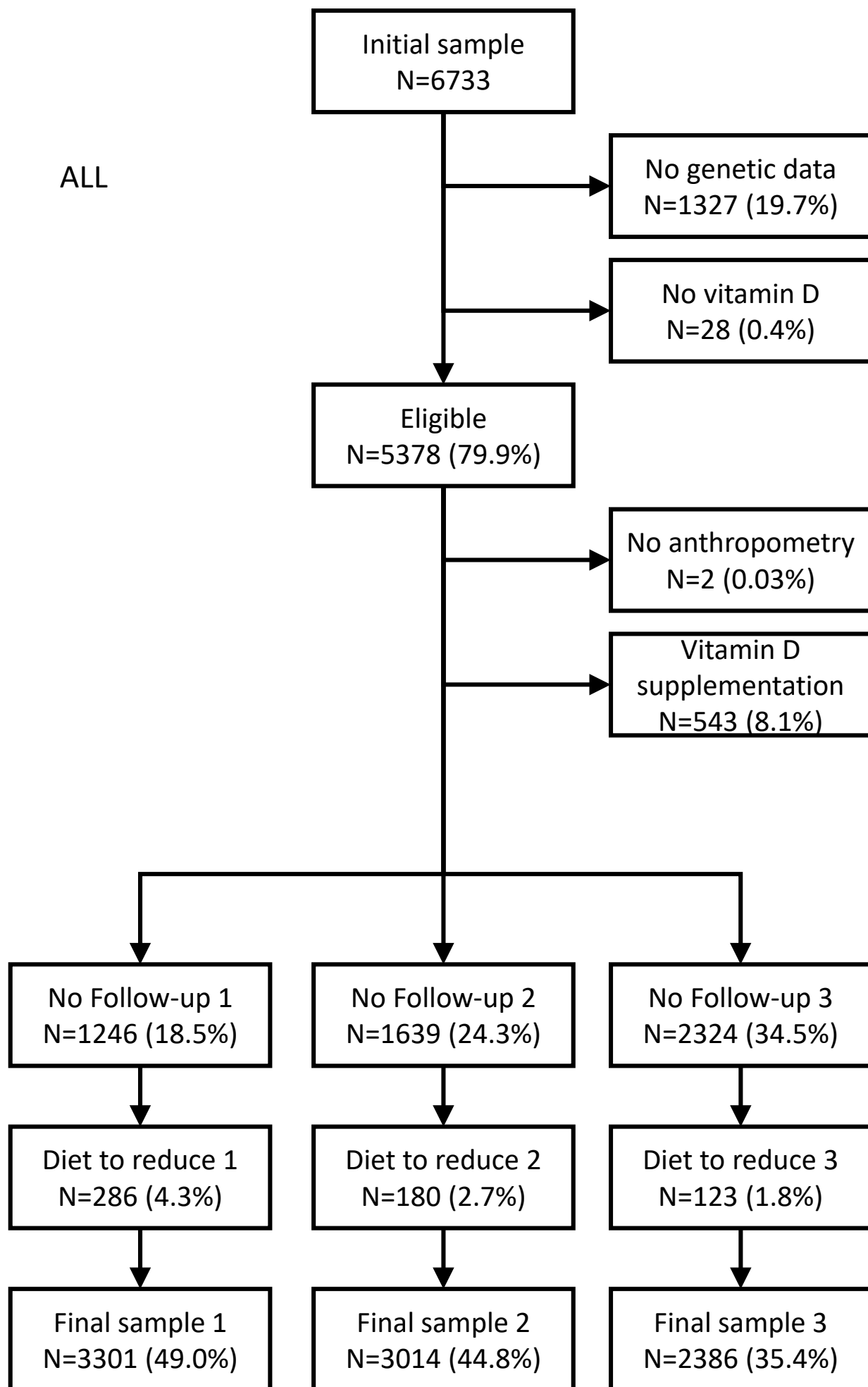

Supplement: Supplementary file 1 [file ijms-23-11100-s001.zip › Figure S2_IJMS.pdf]
